# Machine learning-based model development for predicting risk factors of prolonged intra-aortic balloon pump therapy in patients with coronary artery bypass grafting

**DOI:** 10.1186/s13019-024-02830-8

**Published:** 2024-06-26

**Authors:** Changqing Yang, Peng Zheng, Luo Li, Qian Zhang, Zhouyu Luo, Zhan Shi, Sheng Zhao, Quanye Li

**Affiliations:** 1 Department of Emergency, The Sixth Affiliated Hospital of Nantong University, 2 Xinduxi Road, Yancheng, Jiangsu 224000 China; 2 Department of Emergency, Yancheng Third People’s Hospital, 2 Xinduxi Road, Yancheng, Jiangsu 224000 China; 3 Department of Cardiology, The First Affiliated Hospital of Nanjing Medical University, 300 Guangzhou Road, Nanjing, Jiangsu 210029 China; 4 Department of Cardiovascular Surgery of the First Affiliated Hospital & Institute for Cardiovascular Science, Suzhou Medical College, Soochow University, Soochow University, 899 Pinghai Road, Suzhou, Jiangsu 215123 China; 5 Department of Cardiovascular Surgery, The Sixth Affiliated Hospital of Nantong University, 2 Xinduxi Road, Yancheng, Jiangsu 224000 China; 6 Department of Cardiovascular Surgery, Yancheng Third People’s Hospital, 2 Xinduxi Road, Yancheng, Jiangsu 224000 China; 7 Department of Cardiovascular Surgery, The First Affiliated Hospital of Nanjing Medical University, 300 Guangzhou Road, Nanjing, 210029 Jiangsu China

**Keywords:** Machine learning, Prolonged IABP, Risk factors, Prediction

## Abstract

Machine learning algorithms are frequently used to clinical risk prediction. Our study was designed to predict risk factors of prolonged intra-aortic balloon pump (IABP) use in patients with coronary artery bypass grafting (CABG) through developing machine learning-based models. Patients who received perioperative IABP therapy were divided into two groups based on their length of IABP implantation longer than the 75th percentile for the whole cohort: normal (≤ 10 days) and prolonged (> 10 days) groups. Seven machine learning-based models were created and evaluated, and then the Shapley Additive exPlanations (SHAP) method was employed to further illustrate the influence of the features on model. In our study, a total of 143 patients were included, comprising 56 cases (38.16%) in the prolonged group. The logistic regression model was considered the final prediction model according to its most excellent performance. Furthermore, feature important analysis identified left ventricular end-systolic or diastolic diameter, preoperative IABP use, diabetes, and cardiac troponin T as the top five risk variables for prolonged IABP implantation in patients. The SHAP analysis further explained the features attributed to the model. Machine learning models were successfully developed and used to predict risk variables of prolonged IABP implantation in patients with CABG. This may help early identification for prolonged IABP use and initiate clinical interventions.

**Supplementary Information:**

The online version contains supplementary material available at 10.1186/s13019-024-02830-8.

## Introduction

Short-to-midterm mortality in acute coronary syndrome (ACS) patients complicating cardiogenic shock remains high at rates between 40% and 60% [1–4]. The intra-aortic balloon pumping (IABP), as one of hemodynamic support devices, is implanted into the aorta to temporarily support cardiac output in cardiogenic shock patients [5]. The registry and experimental trials have suggested that it can elevate diastolic blood pressure through promoting forward flow from a high-capacitance reservoir to a low-capacitance vessels, thereby improving coronary and peripheral perfusion and preserving the cardiac function [4, 5]. Despite the supporting evidence for the benefits of IABP, recent IABP-SHOCK II trial (IABP in Cardiogenic Shock II) do not exhibit a beneficial effect of IABP use on 30-day and one-year mortality, which may be associated with IABP-caused complications [6, 7]. Indeed, numerous previous studies have extensively explored the complications related to IABP implantation, such as hemorrhage, limb ischemia, embolization, and thrombocytopenia [8–10]. Additionally, renal function damage is a frequent complication observed in patients undergoing IABP treatment [11]. These complications have been reported to exhibit a positive correlation with the duration of IABP use [10, 12]. However, increased IABP use may impact in a certain degree the length of hospital stay (LOS), the duration of intensive care unit (ICU) stay, hospital costs, and in-hospital death. Accordingly, exploring risk factors for prolonged use of IABP may be of great significance for patients.

Coronary artery bypass grafting (CABG) is the most common heart operation which is performed for treating ACS patients in cardiac surgery centers. During the perioperative period of CABG, these patients complicating cardiogenic shock are frequently required for using IABP support therapy. Some previous studies primarily focus on investigating the effects of IABP implantation timing on clinical outcomes [13, 14]. However, there still are few studies for exploring risk factors of IABP itself use in patients, specifically in CABG individuals. As a result, a highly effective prediction tool for prolonged IABP use in patients is expected to be developed. Machine learning is a novel artificial intelligence-based modeling tool and has been recognized as excellent tool for biomedical research, customized treatment, and computer-aided diagnosis [15]. It is gradually being applied in clinical research and practice to achieve various tasks, such as risk stratification, diagnostic classification, and survival prediction [16]. The aim of this study was to create and evaluate supervised machine learning models to perform risk prediction of prolonged IABP implantation in patients with CABG.

## Methods

### Patient population

This retrospective study was approved by the Ethics Review Committee of The First Affiliated Hospital of Nanjing Medical University (ethics number: 2019-SR-313.A1). Considering the nature of the retrospective study, patients’ informed consent was waived by the Ethics Review Committee of the hospital. Between January 2015 to December 2019, all adult patients who underwent an isolated CABG surgery and received perioperative IABP support therapy were enrolled into this study in a way of the chronological mode. Standard median sternotomy, cardiopulmonary bypass (CPB), and aortic cross-clamp were applied in all patients. The timing of IABP implantation in patients was evaluated by the entire medical team. The indications for IABP insertion in all patients were: (1) blood pressure decreasing progressively under the therapy of two vasoactive drugs, (2) mean arterial pressure < 50 mmHg, (3) cardiac index (CI) < 2.2 L/(min·m^2^), (4) mean arterial pressure < 50 mmHg, and (5) urine volume < 0.5 mL/(kg·h). In this study, all patients who met the inclusion criteria were enrolled during the study period, and a total of 143 patients were collected.

### Data collection

Patients were characterized by 46 rapid available preoperative variables (including demographics, comorbidities, coronary artery lesion and angiography, echocardiography, electrocardiogram (ECG), and laboratory indicators). Moreover, information on continuous renal replacement therapy (CRRT) and tracheotomy in patients during the IABP insertion was collected. LOS, length of ICU, in-hospital mortality, and hospital costs were recorded. All data were input and audited by experienced physicians using the electronic medical record (EMR) system of the hospital. Some previous publications defined prolonged duration of IABP to be between 2 and 14 days; however, this definition has not been adopted by all investigators. In this study, we stratified the patients into the following three groups based on the length of IABP therapy (LOIT): the lowest quartile (25th quartile), the median (25th–75th quartiles), and the highest quartile (75th quartile). In this study, we defined the 75th quartile of LOIT (10 days) as the demarcation line between normal and prolonged periods: normal LOIT (Nor-LOIT, ≤ 10 days) and prolonged LOIT (Pro-LOIT, > 10 days) groups.

### Feature selection for modeling

The Boruta algorithm is used to evaluate the importance of each variable in a circular manner, comparing the importance of original variables and its shadow variables in each iteration round. If the importance of original variable is significantly higher than that of the shadow variable, it is considered important. Conversely, if original variable is considerably less important than its shadow counterpart, it is deemed unimportant. In this study, the Boruta algorithm was performed to select the most crucial features associated with clinical outcome from the collected variables. Subsequently, these variables were employed to the construction and development of the models, which could effectively avoid overfitting and optimize hyperparameters.

### Machine learning models development and validation

The original dataset collected in this study was randomly separated into a training (90%) dataset and an internal validation (10%) dataset. The training dataset was employed to train the models, while the validation dataset was used for the evaluation and selection of the models. During this process, 10-fold cross-validation was performed. In this study, seven machine learning models were developed to predict the risk factors of prolonged LOIT in patients with CABG surgery, including logistic regression, LightGBM, Gaussian Naive Bayes (GNB), multi-layer perceptron neural network (MLP), k-nearest neighbors (KNN), support vector machine (SVM), and Complement Naive Bayes (CNB).

**Fig. 1:**
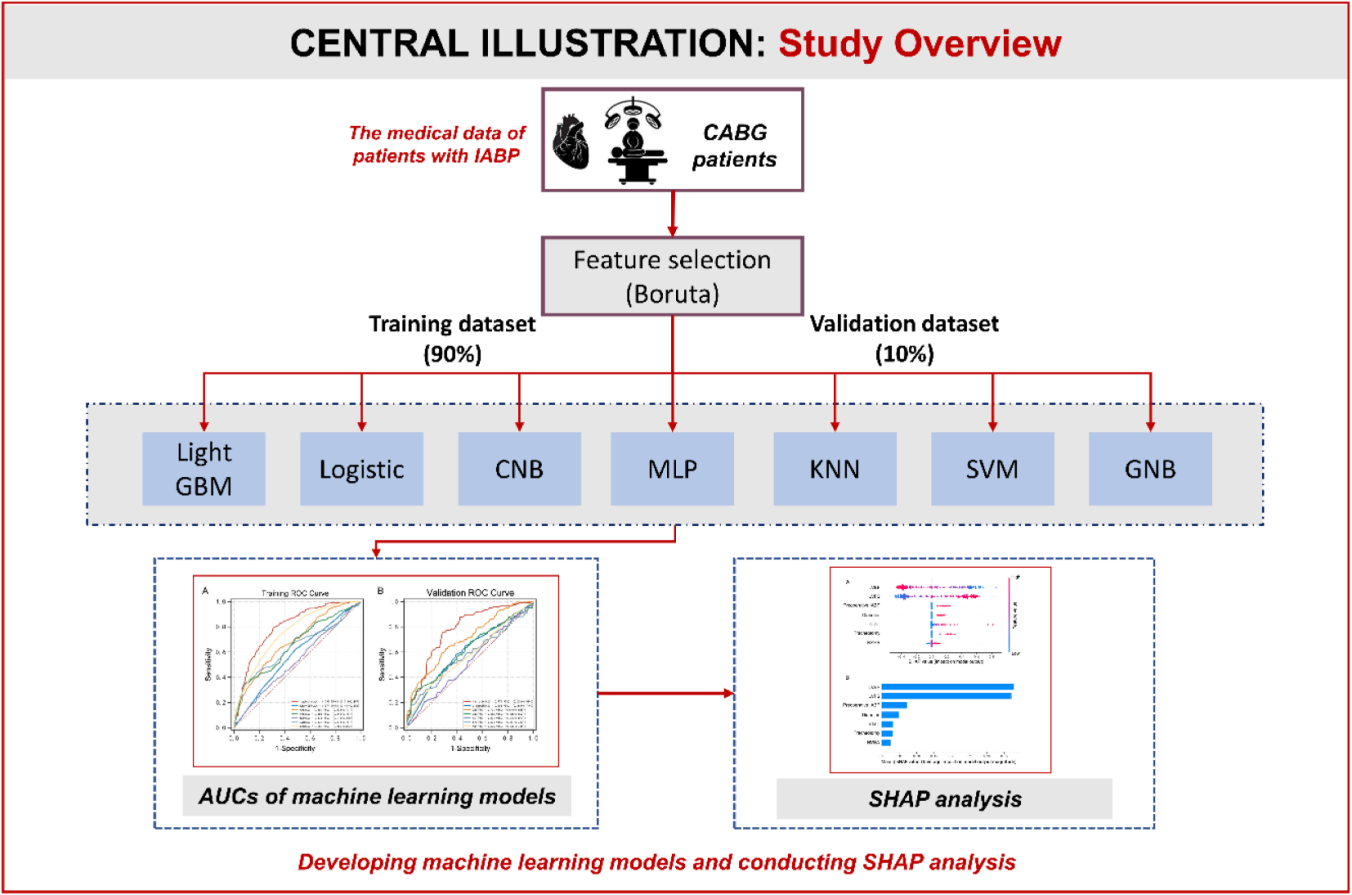
Overall flowchart of the study. IABP, intra-aortic balloon pump; CABG, coronary artery bypass grafting; GNB, Gaussian Naive Bayes; MLP, multi-layer perceptron neural network; KNN, k-nearest neighbors; SVM, support vector machine; CNB, Complement Naive Bayes; AUC, area under the receiver operating characteristic curve; SHAP, Shapley Additive exPlanations

Then, performance of machine learning models was measured using the area under the receiver operating characteristic curve (AUC) with associated 95% confidence interval (CI). The accuracy (ACC), sensitivity, specificity, positive predictive value (PPV), negative predictive value (NPV), and F1-score were calculated for further evaluation of the model performance. The calibration plot was visualized to assess the model’s calibration by calculating the Brier score, where a smaller Brier value indicates higher accuracy of the model. This implies that the discrepancy between predicted outcomes and actual clinical practice outcomes is minimized. The final predictive model in this study was chosen based on its superior performance. Figure 1***shows the flowchart of this study.***

### Shapley Additive exPlanations (SHAP) application

The SHAP method was applied to enhance the interpretability of the final predictive model, and the SHAP summary plot was used to illustrate the influence of model features. The SHAP dependence plot was used to analyze the importance of individual features affecting model output. The SHAP force plot was utilized to visually represent the impact of the features on the final model in individual patients.

### Statistical analysis

Continuous variables were presented as mean ± standard deviations (SD) or median (interquartile spacing). Continuous variables with a normalized distribution among two groups were analyzed using Students’ t-test. Continuous variables with non-normalized distribution were analyzed using the Mann-Whitney U test. Categorical variables were presented as numerical values (proportions) and analyzed using the chi-square test. All statistical analysis in this study was conducted using IBM SPSS statistics software (version 25.0, IBM Corp., Armonk, New York, USA). The values of *p* < 0.05 represented a significant statistical difference.

## Results

### Baseline characteristics of patients

A total of 143 patients who underwent CABG and received perioperative IABP support therapy were enrolled in this study, comprising 116 males (81.12%), with a median age of 66 years and 19 mortalities (13.29%). Among these patients, 56 cases (39.16%) were included into the Pro-LOIT group, with 46 males (82.14%) and eight mortalities (14.29%). The significant differences were observed between the two groups regarding diabetes, total cholesterol (TC), high-density lipoprotein cholesterol (HDL-C), fast blood glucose, cardiac troponin T (cTnT), New York Heart Association (NYHA) classification, left ventricular end-systolic dimension (LVDs), left ventricular ejection fraction (LVEF), left ventricular fraction shortening (LVFS), tracheotomy, preoperative IABP insertion, LOS, length of ICU, and hospital costs. The detail content is shown in Table 1.

**Table 1:** Baseline characteristics of patients in the two groups

Variables		Nor-LOIT (*N* = 87)	Pro-LOIT (*N* = 56)	Statistical	*P*-value
**Age (year)**		65.00 (60.00, 71.00)	66.00 (58.00, 71.00)	0.14	0.890
**Gender (%)**	**Male**	70 (80.46)	46 (82.14)	0.06	0.802
	**Female**	17 (19.54)	10 (17.86)		
**Alcohol history (%)**	**No**	60 (68.97)	40 (71.43)	0.10	0.754
	**Yes**	27 (31.03)	16 (28.57)		
**Smoking history (%)**	**No**	49 (56.32)	27 (48.21)	0.90	0.343
	**Yes**	38 (43.68)	29 (51.79)		
**Overweight (%)**	**No**	44 (50.57)	25 (44.64)	0.48	0.488
	**Yes**	43 (49.43)	31 (55.36)		
**LM stenosis ≥ 70%**	**No**	63 (72.41)	35 (62.50)	1.55	0.213
	**Yes**	24 (27.59)	21 (37.50)		
**Coronary occlusion number (%)**	**0**	29 (33.33)	20 (35.71)	3.38	0.336
	**1**	30 (34.48)	24 (42.86)		
	**2**	21 (24.14)	11 (19.64)		
	**3**	7 (8.05)	1 (1.79)		
**LAD occlusion (%)**	**No**	56 (64.37)	41 (73.21)	1.22	0.269
	**Yes**	31 (35.63)	15 (26.79)		
**LCX occlusion (%)**	**No**	59 (67.82)	44 (78.57)	1.96	0.162
	**Yes**	28 (32.18)	12 (21.43)		
**AMI history (%)**	**No**	39 (44.83)	16 (28.57)	3.80	0.051
	**Yes**	48 (55.17)	40 (71.43)		
**NYHA classification (%)**	**I**	19 (21.84)	2 (3.57)	12.72	**0.005**
	**II**	40 (45.98)	23 (41.07)		
	**III**	25 (28.74)	29 (51.79)		
	**IV**	3 (3.45)	2 (3.57)		
**Hypertension (%)**	**No**	28 (32.18)	18 (32.14)	0	0.996
	**Yes**	59 (67.82)	38 (67.86)		
**Stroke history (%)**	**No**	68 (78.16)	46 (82.14)	0.33	0.563
	**Yes**	19 (21.84)	10 (17.86)		
**Diabetes (%)**	**No**	57 (65.52)	26 (46.43)	5.10	**0.024**
	**Yes**	30 (34.48)	30 (53.57)		
**Chronic kidney disease (%)**	**No**	82 (94.25)	51 (91.07)	0.53	0.467
	**Yes**	5 (5.75)	5 (8.93)		
**Chronic liver disease (%)**	**No**	86 (98.85)	55 (98.21)	0.10	0.752
	**Yes**	1 (1.15)	1 (1.79)		
**Chronic lung disease (%)**	**No**	85 (97.70)	53 (94.64)	0.94	0.331
	**Yes**	2 (2.30)	3 (5.36)		
**Atrial fibrillation (%)**	**No**	79 (90.80)	50 (89.29)	0.09	0.765
	**Yes**	8 (9.20)	6 (10.71)		
**Atrioventricular block (%)**	**No**	81 (93.10)	49 (87.50)	1.29	0.255
	**Yes**	6 (6.90)	7 (12.50)		
**Abnormal Q wave (%)**	**No**	62 (71.26)	43 (76.79)	0.53	0.466
	**Yes**	25 (28.74)	13 (23.21)		
**Abnormal ST-T segment (%)**	**No**	12 (13.79)	9 (16.07)	0.14	0.707
	**Yes**	75 (86.21)	47 (83.93)		
**Premature contraction (%)**	**No**	68 (78.16)	44 (78.57)	0	0.954
	**Yes**	19 (21.84)	12 (21.43)		
**Bundle branch block (%)**	**No**	79 (90.80)	46 (82.14)	2.32	0.127
	**Yes**	8 (9.20)	10 (17.86)		
**RV5 + SV1 (mV)**		2.15 (1.77,2.67)	2.20 (1.82,2.61)	-0.51	0.610
**QRS (ms)**		100.00 (93.00, 105.80)	103.00 (97.00, 105.80)	-1.35	0.176
**QT internal (ms)**		397.47 (379.00,422.00)	397.47 (385.00,421.74)	0.49	0.628
**HR (bmp)**		73.00 (66.00, 80.00)	73.00 (64.00, 85.00)	-0.25	0.802
**Preoperative IABP**	**No**	59 (67.82)	22 (39.29)	11.29	**< 0.001**
	**Yes**	28 (32.18)	34 (60.71)		
**CRRT (%)**	**No**	66 (75.86)	37 (66.07)	1.62	0.203
	**Yes**	21 (24.14)	19 (33.93)		
**Tracheotomy (%)**	**No**	80 (91.95)	43 (76.79)	6.52	**0.011**
	**Yes**	7 (8.05)	13 (23.21)		
**PAP (mmHg)**		30.00 (26.00,35.00)	30.00 (25.00,36.00)	0.50	0.615
**LVDs (mm** ^**3**^ **)**		35.00 (31.00, 43.00)	39.00 (35.00, 43.00)	-2.05	**0.040**
**LVDd (mm** ^**3**^ **)**		51.00 (47.00, 56.00)	52.00 (50.00, 56.00)	-1.58	0.114
**LVEF (%)**		58.00 (44.60, 63.00)	49.10 (42.90, 59.00)	2.34	**0.019**
**LVFS (%)**		31.10 (22.60, 34.00)	25.00 (21.40, 31.60)	2.29	**0.022**
**cTnT (ng/mL)**		89.49 (41.53, 419.70)	275.48 (47.95, 1845.00)	-2.36	**0.018**
**CK-MB (ng/mL)**		11.01 (5.70, 16.20)	11.02 (6.00, 21.00)	-1.10	0.270
**BNP (pg/mL)**		1569.50 (605.00,4131.81)	2081.19 (829.36,5089.00)	-1.24	0.215
**BUN (mmol/L)**		6.39 (5.30, 7.88)	7.71 (5.27, 9.46)	-1.37	0.172
**Scr (µmol/L)**		77.30 (66.60, 94.50)	87.10 (65.10, 113.40)	-1.41	0.160
**Fast blood glucose (mmol/L)**		5.57 (4.75, 6.74)	6.16 (5.30, 9.31)	-2.23	**0.026**
**ALB (g/L)**		36.34 ± 3.69	35.13 ± 3.94	1.86	0.064
**TC (mmol/L)**		3.82 (3.27, 5.01)	3.73 (2.94, 4.37)	1.94	0.052
**TG (mmol/L)**		1.25 (0.94, 1.68)	1.30 (0.96, 2.01)	-0.81	0.418
**HDL-C (mmol/L)**		0.92 (0.86, 1.06)	0.90 (0.74, 0.99)	2.44	**0.015**
**LDL-C (mmol/L)**		2.37 (1.95, 3.19)	2.27 (1.79, 2.65)	1.89	0.058
**LOS (d)**		22.00 (18.00, 30.00)	30.00 (25.00, 43.00)	-4.04	**< 0.001**
**Length of ICU (d)**		9.00 (6.00, 13.00)	18.00 (14.00, 26.00)	-5.98	**< 0.001**
**Death (%)**	**No**	76 (87.36)	48 (85.71)	0.08	0.778
	**Yes**	11 (12.64)	8 (14.29)		
**Hospital costs (RMB)**		264,855 (217,760, 330,657)	396,730 (310,920, 524,352)	-5.57	< 0.001

*Note* preoperative IABP indicates IABP insertion before cardiac surgery. Overweight indicates that body mass index is more than 24.0 kg/m^2^. Coronary occlusion indicates that coronary lesion stenosis is more than 90%. Chronic liver disease indicates chronic hepatitis and chronic liver cirrhosis. Chronic lung disease indicates chronic bronchitis, chronic obstructive pulmonary disease, and emphysema. Continuous variables were presented as mean ± standard deviations (SD) or median (interquartile spacing). Categorical variables were presented as numerical values and proportions. LM, left main; LAD, left anterior descending artery; LCX, left circumflex artery; NYHA, New York Heart Association; AMI, acute myocardial infarction; HR, heart rate; IABP, intra-aortic balloon pump; CRRT, continuous renal replacement therapy; PAP, pulmonary arterial pressure; LVDs, left ventricular end-systolic diameter; LVDd, left ventricular end-diastolic diameter; LVEF, left ventricular ejection fraction; LVFS, left ventricular fraction shortening; cTnT, cardiac troponin T; CK-MB, creatine kinase isoenzyme; BNP, brain natriuretic peptide; BUN, blood urea nitrogen; Scr, serum creatinine; ALB, albumin; TC, total cholesterol; TG, triglyceride; HDL-C, high-density lipoprotein cholesterol; LDL-C, low-density lipoprotein cholesterol; LOS, length of hospital stay; ICU, intensive care unit

### Feature selection based on the Boruta algorithm

**Fig. 2:**
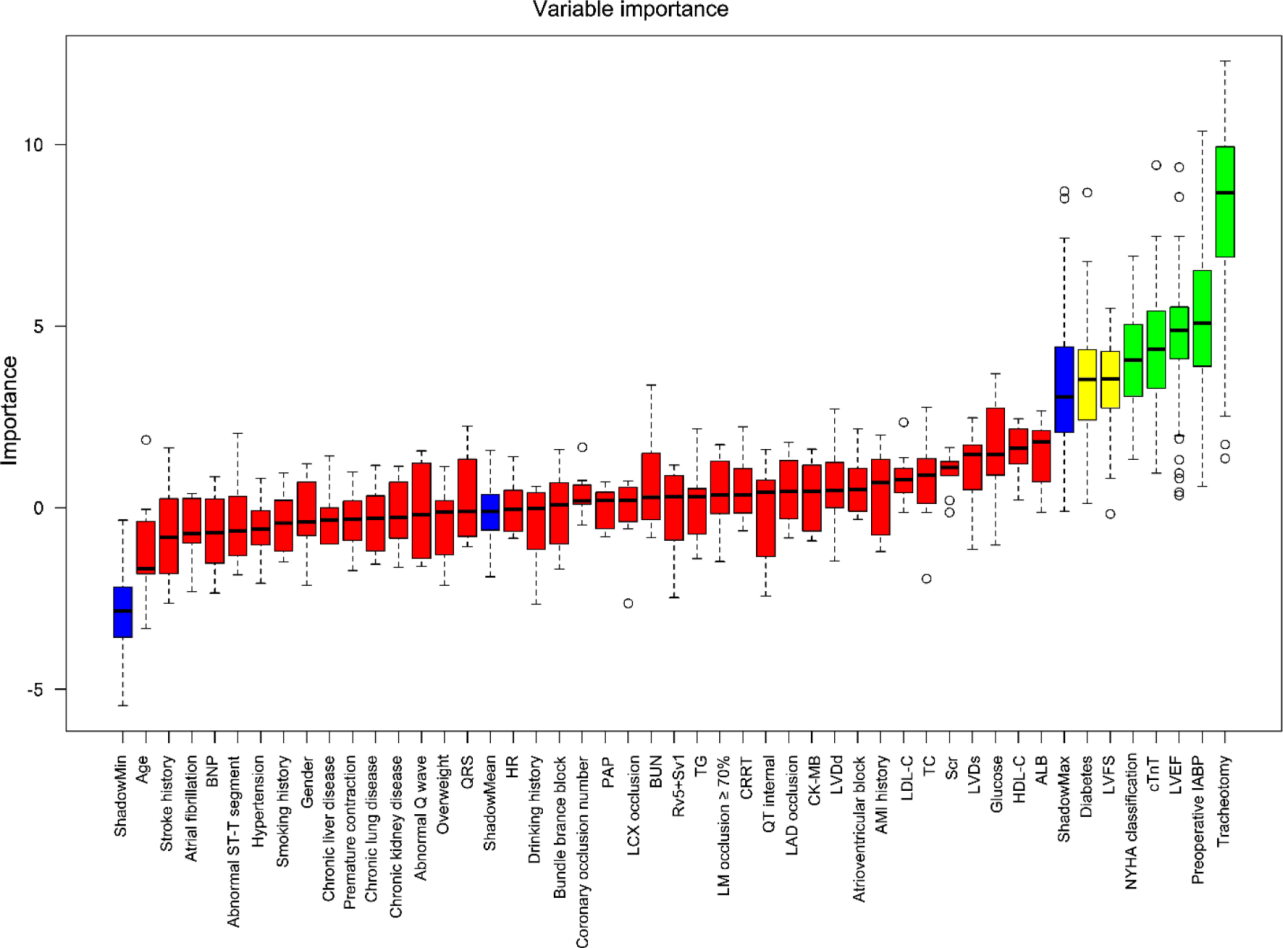
The Boruta algorithm for feature selection. When importance of feature exceeds the value of ShadowMax, these features are selected for developing models

The Boruta algorithm was employed to identify the most crucial variables associated with Pro-LOIT in patients. Ultimately, 7 variables were identified and used to develop machine learning models. This selection process was effective in avoiding overfitting and optimizing hyperparameters. However, the selection based on the Boruta algorithm did not imply that importance of the variables was analyzed in this study. These selected variables included tracheotomy, preoperative IABP, LVEF, cTnT, NYHA classification, LVFS, and diabetes. The corresponding results are shown in Fig. 2.

### Models’ performance in predicting prolonged LOIT risk

**Fig. 3:**
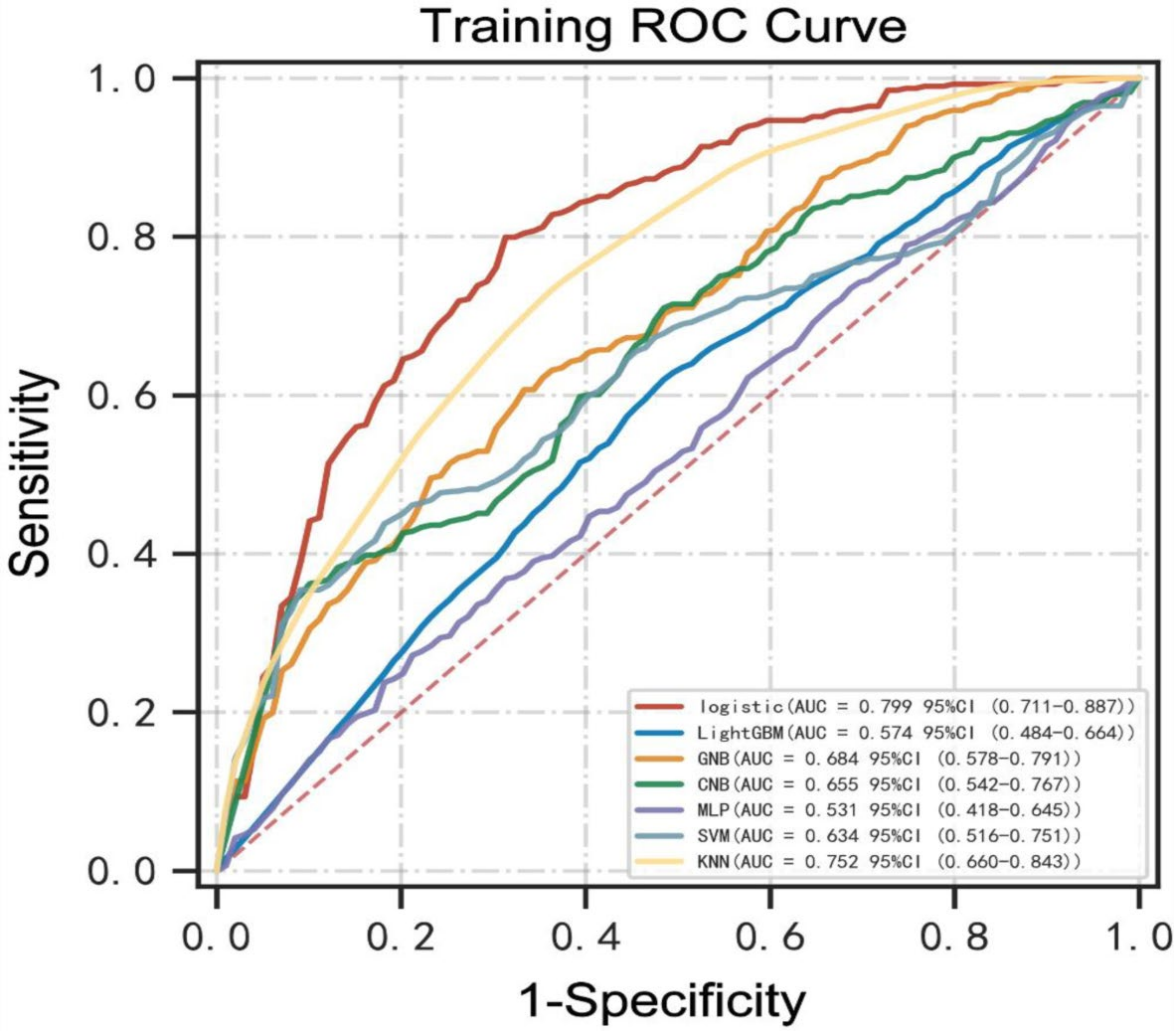
The receiver operating characteristic curves (ROCs) of machine learning models in the training set. GNB, Gaussian Naive Bayes; MLP, multi-layer perceptron neural network; KNN, k-nearest neighbors; SVM, support vector machine; CNB, Complement Naive Bayes; AUC, area under the receiver operating characteristic curve

**Fig. 4:**
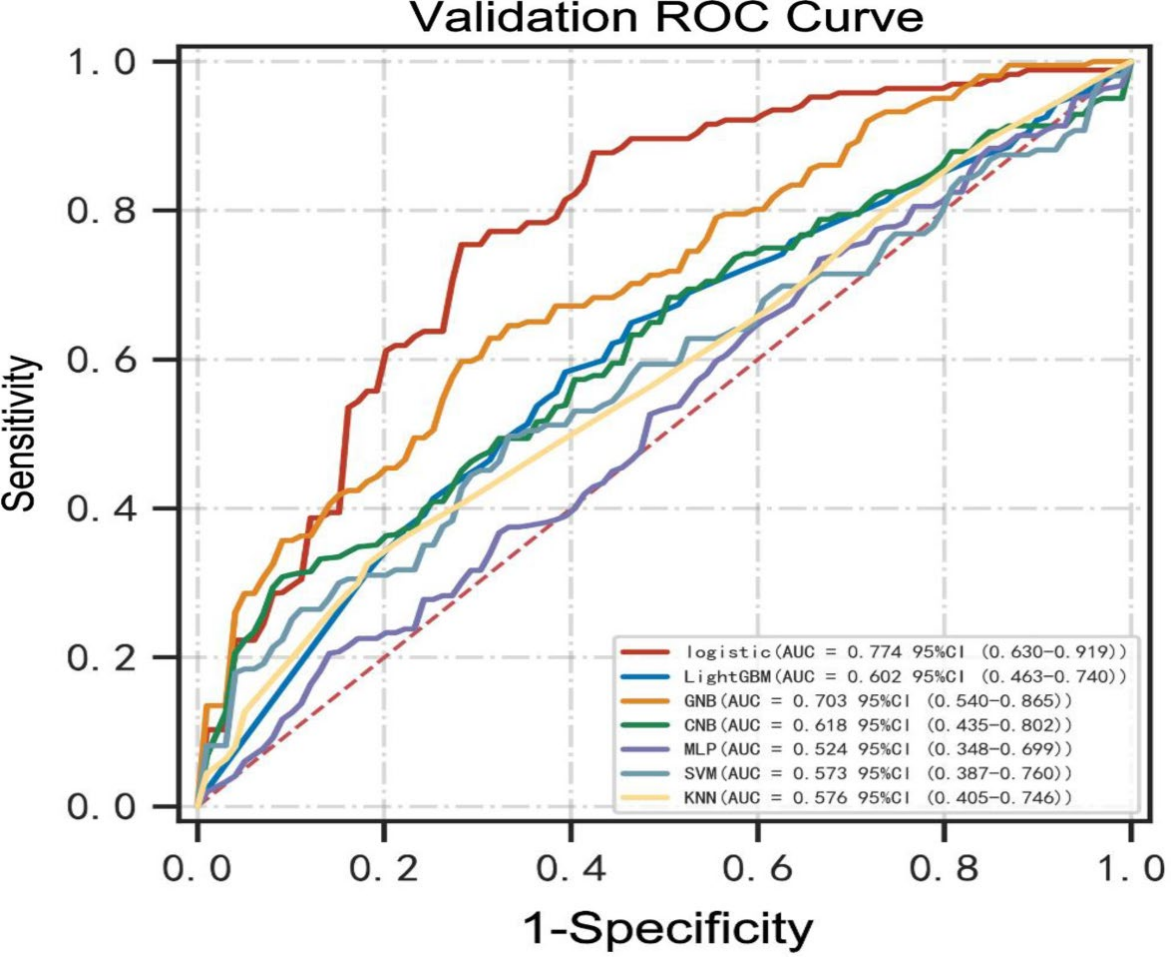
The receiver operating characteristic curves (ROCs) of machine learning models in the validation set. GNB, Gaussian Naive Bayes; MLP, multi-layer perceptron neural network; KNN, k-nearest neighbors; SVM, support vector machine; CNB, Complement Naive Bayes; AUC, area under the receiver operating characteristic curve

**Fig. 5:**
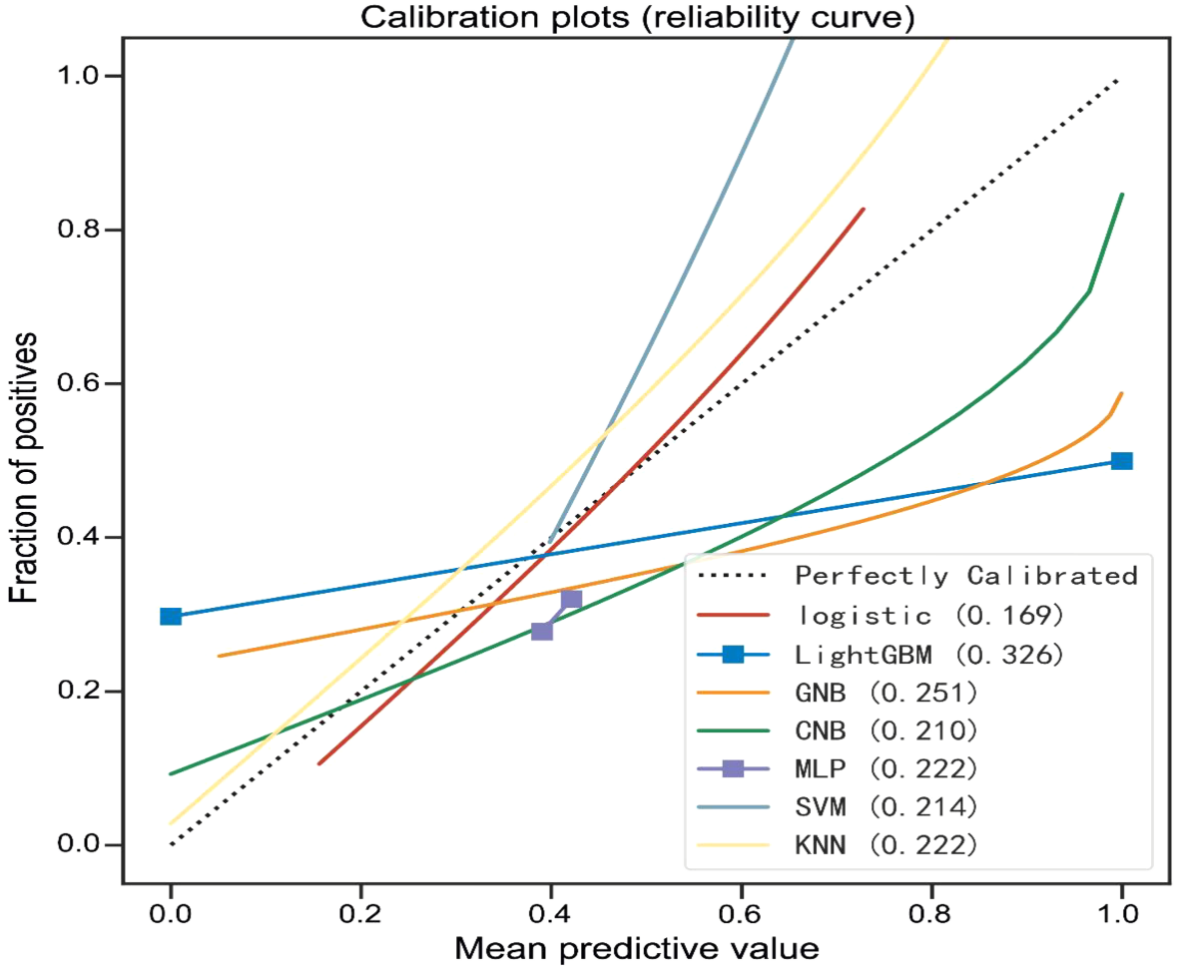
The calibration plots (reliability curves) of machine learning models. GNB, Gaussian Naive Bayes; MLP, multi-layer perceptron neural network; KNN, k-nearest neighbors; SVM, support vector machine; CNB, Complement Naive Bayes

The predictive performance of machine learning models is illustrated in Figs. 3–4; Tables 2 and 3. Our findings showed that the models had various abilities to predict the risk factors associated with prolonged LOIT in patients. Compared to other models, the logistic regression model exhibited an excellent predictive performance due to its highest AUC (0.799, 95%CI: 0.711–0.887, Fig. 3) in the training set and (0.774, 95%CI: 0.630–0.919, Fig. 4) in the validation set. Furthermore, the highest ACC, sensitivity, and F1-score in the two datasets of the logistic regression model were found (Tables 2 and 3). The calibration curve plotting was created and is shown in Fig. 5. The predictive probability of the logistic regression model was the closest to clinical practice outcome. Based on these findings, we eventually considered the logistic regression model as the predictive model for prolonged LOIT.

**Table 2:** The parameters of models in the training set

Models	AUC	ACC	Sensitivity	Specificity	PPV	NPV	F1-score
**Logistic regression**	0.80 (0.71–0.89)	0.74 (0.73–0.76)	0.81 (0.77–0.85)	0.71 (0.67–0.76)	0.65 (0.62–0.67)	0.84 (0.82–0.86)	0.72 (0.70–0.73)
**LightGBM**	0.57 (0.48–0.66)	0.58 (0.53–0.63)	0.58 (0.37–0.78)	0.60 (0.41–0.78)	NA	0.69 (0.63–0.74)	NA
**GNB**	0.68 (0.58–0.79)	0.67 (0.64–0.69)	0.65 (0.57–0.73)	0.69 (0.61–0.77)	0.58 (0.54–0.63)	0.75 (0.72–0.78)	0.60 (0.58–0.62)
**CNB**	0.65 (0.54–0.77)	0.66 (0.62–0.69)	0.53 (0.41–0.65)	0.76 (0.65–0.88)	NA	0.71 (0.68–0.74)	NA
**MLP**	0.53 (0.42–0.65)	0.56 (0.51–0.61)	0.66 (0.50–0.83)	0.51 (0.32–0.69)	0.49 (0.43–0.55)	0.71 (0.66–0.76)	0.53 (0.47–0.59)
**SVM**	0.63 (0.52–0.75)	0.68 (0.65–0.70)	0.45 (0.37–0.53)	0.85 (0.77–0.92)	0.68 (0.61–0.75)	0.69 (0.66–0.72)	0.52 (0.50–0.54)
**KNN**	0.75 (0.66–0.84)	0.68 (0.66–0.70)	0.75 (0.68–0.83)	0.65 (0.56–0.73)	0.70 (0.61–0.78)	0.69 (0.67–0.72)	0.71 (0.67–0.75)

AUC, area under the receiver operating characteristic curve; ACC, accuracy; PPV, positive prediction value; NPV, negative prediction value; GNB, Gaussian Naive Bayes; CNB, complement Naive Bayes; MLP, multi-layer perceptron neural network; SVM, support vector machine; KNN, k-nearest neighbors. NA: Not applicable. Results are shown as value (95% CI)

**Table 3:** The parameters of models in the validation set

Models	AUC	ACC	Sensitivity	Specificity	PPV	NPV	F1-score
**Logistic regression**	0.77 (0.63–0.92)	0.68 (0.65–0.71)	0.84 (0.77–0.90)	0.70 (0.64–0.76)	0.59 (0.53–0.64)	0.77 (0.72–0.82)	0.69 (0.64–0.74)
**LightGBM**	0.60 (0.46–0.74)	0.58 (0.53–0.62)	0.61 (0.38–0.83)	0.65 (0.49–0.81)	NA	0.68 (0.58–0.79)	NA
**GNB**	0.70 (0.54–0.87)	0.66 (0.61–0.71)	0.64 (0.57–0.72)	0.77 (0.69–0.85)	0.55 (0.49–0.62)	0.74 (0.70–0.78)	0.59 (0.53–0.65)
**CNB**	0.62 (0.44–0.80)	0.58 (0.54–0.63)	0.64 (0.50–0.78)	0.67 (0.51–0.84)	NA	0.65 (0.61–0.69)	NA
**MLP**	0.52 (0.35–0.70)	0.54 (0.46–0.61)	0.66 (0.46–0.87)	0.57 (0.38–0.76)	0.44 (0.35–0.53)	0.71 (0.61–0.80)	0.49 (0.36–0.63)
**SVM**	0.57 (0.39–0.76)	0.62 (0.57–0.68)	0.53 (0.42–0.63)	0.74 (0.63–0.84)	0.53 (0.45–0.60)	0.64 (0.59–0.70)	0.50 (0.45–0.56)
**KNN**	0.58 (0.41–0.75)	0.63 (0.60–0.67)	0.56 (0.40–0.72)	0.66 (0.48–0.84)	0.55 (0.36–0.73)	0.64 (0.60–0.68)	0.42 (0.31–0.54)

AUC, area under the receiver operating characteristic curve; ACC, accuracy; PPV, positive prediction value; NPV, negative prediction value; GNB, Gaussian Naive Bayes; CNB, complement Naive Bayes; MLP, multi-layer perceptron neural network; SVM, support vector machine; KNN, k-nearest neighbors. NA: Not applicable. Results are shown as value (95% CI)

### The logistic regression model explanation and application

**Fig. 6:**
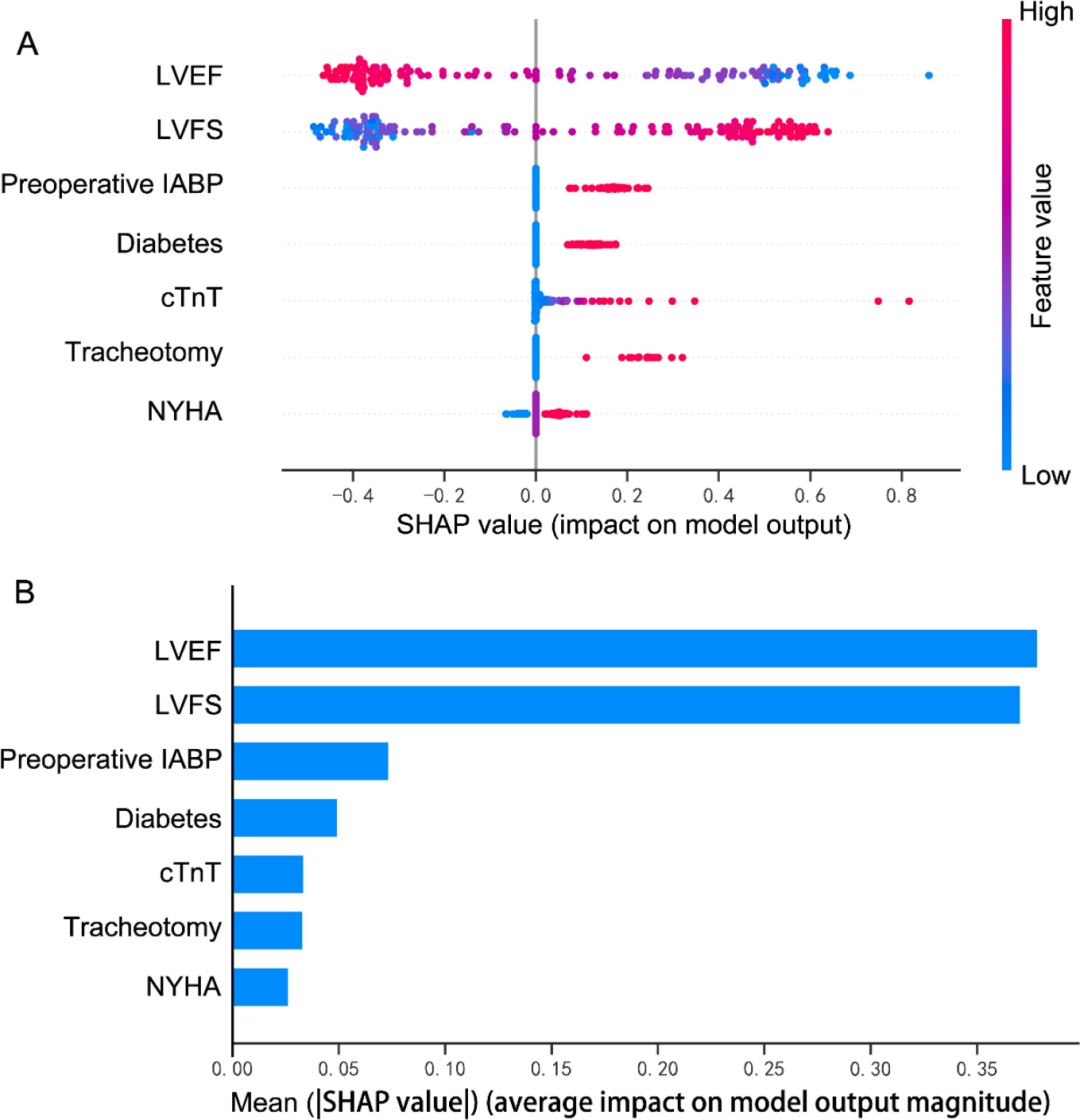
The logistic regression model’s interpretation based on the SHAP. **(A)** The importance ranking of the top 7 variables according to the mean (|SHAP value|). **(B)** The importance ranking of the top 7 risk factors. The higher SHAP value of a feature is shown, the higher risk of prolonged LOIT the patient would have. The red part in feature value represents higher value

**Fig. 7:**
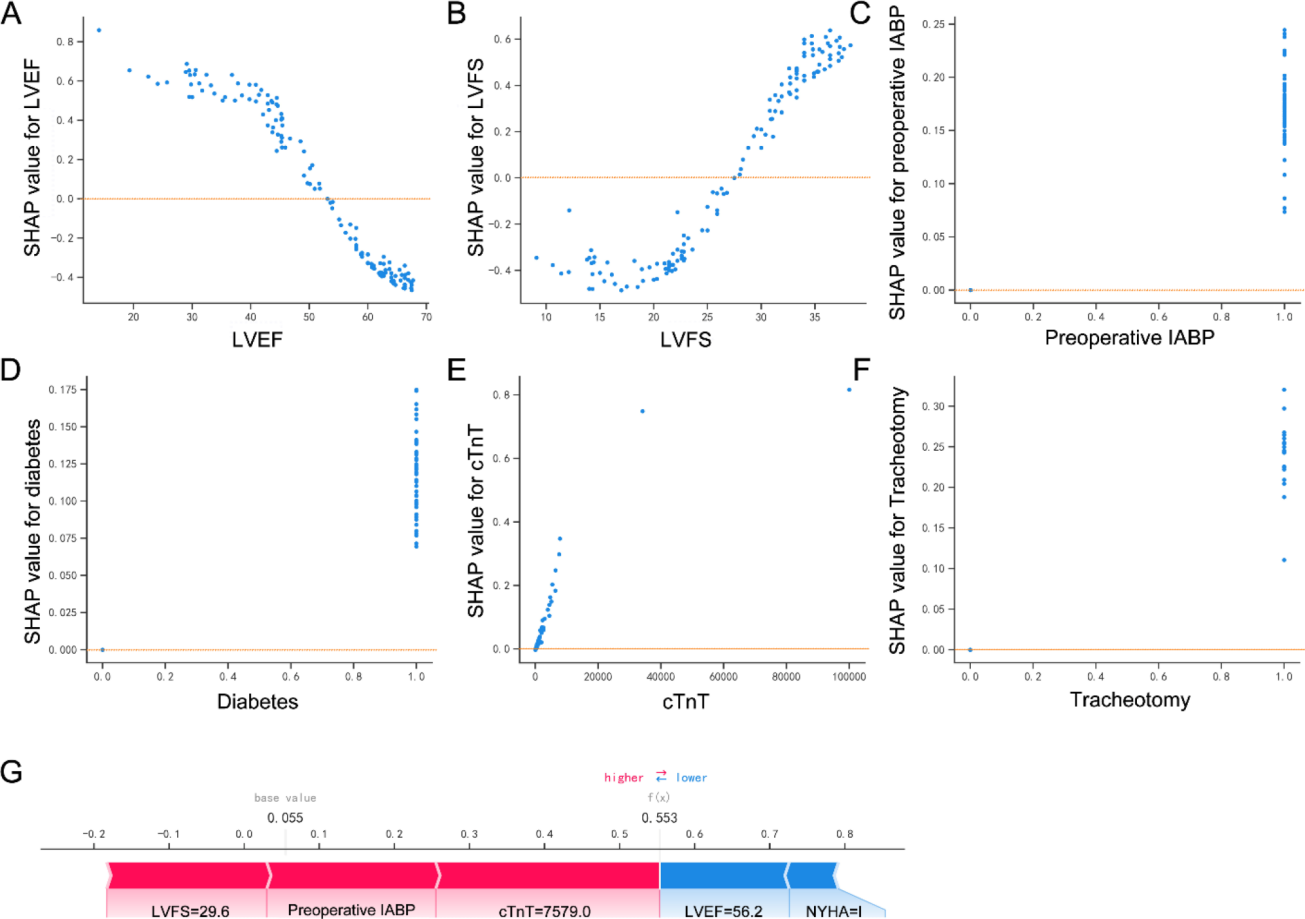
The SHAP dependency plot for the top 6 clinical features contributing to the logistic regression model and interpretation of model prediction results with the one sample. (**A**-**F**) LVEF, LVFS, preoperative IABP, diabetes, cTnT, and NYHA. (**G**) Model predictions by randomly drawing a single sample from the validation cohort

The feature importance for the logistic regression model was analyzed using the SHAP value. This showed the greatest discriminatory capacity in the validation cohort. According to the obtained SHAP value, Fig. 6A exhibits the weight of 7 clinical variables and Fig. 6B provides an overview of the impact (positive or negative aspects) of factors on the logistic regression model. Subsequently, the correlation between variables and the risk factors associated with prolonged LOIT is displayed in Fig. 7A-F, with the positive and negative association. Subsequently, we randomly selected one patient from the validation cohort to exhibit a visual interpretation for an individual patient (Fig. 7G). The logistic regression model predicted the probability of prolonged LOIT in patient to be 55.30%. The result indicated that serum cTnT of 7579.0 pg/L, preoperative IABP use, and LVFS of 29.6% were the top three contributors to this prediction.

## Discussion

In this study, we developed seven machine learning models using selected clinical crucial features to identify the risk factors associated with prolonged LOIT in patients with CABG surgery. Compared to other models, the logistic regression model had the most well predictive performance, which could be confirmed by its highest AUC, ACC, sensitivity, and F1-score, as well as an excellent calibration. Additionally, the SHAP analysis was constructed to exhibit the importance of the variables and how particular compound substructures influence prolonged LOIT in CABG patients in the created logistic regression model. Our result revealed that LVEF, LVFS, preoperative IABP, diabetes, and cTnT were the top five most important variables contributing to the logistic regression model. Finally, the SHAP personal analysis was used to facilitate the individualized predictions.

With the rise of artificial intelligence, machine learning algorithms are expected to become a crucial tool to optimize risk prediction and clinical assessment system [16]. Machine learning models based on artificial intelligence have been successfully developed in the field of perioperative medicine for risk stratification, prediction of intraoperative events, and intensive care medicine [17]. The models could help clinicians improve clinical outcomes by accurately predicting complications and suggesting optimal treatment strategies in real-time [17]. The development of several machine learning-based models has enabled the prediction of perioperative outcomes in patients undergoing cardiac surgeries [18–21]. However, there is a lack of studies on the prediction of risk factors associated with prolonged LOIT in patients undergoing CABG surgery. Furthermore, the development of personalized systems is imperative for accurately predicting outcomes among specific operator groups, which highlights the importance of machine learning models [22, 23]. The aim of personalized medicine has been to make models match the individual across multiple scales to solve clinical issues. During the development of models, it is imperative to emphasize the importance of conducting selection of characteristic variables prior to model development, which is beneficial for identifying optimal parameters and avoiding the model overfitting. Then, the SHAP analysis was used to further demonstrate the weights assigned by the model to relevant factors. The individual explanations generated by SHAP analysis help doctors’ comprehension of why the model provided specific recommendations for high-risk decisions.

To further confirm how factors contribute to the model, we calculated SHAP feature importance and feature effects. The LVEF and LVFS of patient are crucial echocardiographic indicators that reflect the systolic function of left ventricle. A decrease in the parameters indicates impaired left ventricular systolic function. A study revealed a close relationship between the impact of IABP on mortality and the severity of cardiogenic shock, suggesting that cardiac function may affect clinical consequences in patients [24]. Another clinical study investigated the association between preoperative cardiac function, including left systolic function, and perioperative IABP use in patients undergoing elective off-pump coronary artery bypass surgery [25]. Compared to those with normal cardiac function, patients with reduced left ventricular systolic function received IABP support therapy more frequently during the perioperative period of cardiac surgery [25]. Patients with left ventricular dysfunction are more prone to hemodynamic disturbances and related complications during the perioperative period, and therefore may require ventricular assist support or extended support duration. In our study, the LVEF and LVFS of patients in the Pro-LOIT group were lower than those in the Nor-LOIT group, indicating a reduced cardiac function in the prolonged IABP group. The SHAP analysis further identified LVEF and LVFS as the most crucial variables affecting the outcome of prolonged IABP utilization within the logistic regression model in our study. These findings underscore the importance of monitoring left ventricular systolic parameters in patients. Due to the superior sensitivity and specificity of serum cTnT levels as a diagnostic marker, it has been become the current standard laboratory determinant for myocardial injury [26]. Previous studies have shown serum cTnT as a prognostic variable in cardiac or noncardiac surgery, with an elevated serum cTnT levels being related to postoperative complications and mortality following surgeries [27–29]. Our model identified serum cTnT levels as crucial variable of prolonged IABP implantation in patients. This suggests that there is a positive relationship between cardiac injury and IABP support therapy.

Previous studies have extensively explored the influence of the timing of IABP implantation on postoperative clinical outcomes, including mortality, LOS, ICU, and complications [13, 30–32]. Our study revealed that preoperative IABP implantation was an important risk factor affecting the LOIT of patients. Preoperative IABP implantation frequently implies that when ACS patients with cardiogenic shock are unable to immediately undergo CABG surgery, and IABP is only considered a temporary support device to improve clinical symptoms of patient. Previous study indicates that patients receiving preoperative IABP use have a higher risk of cardiac dysfunction, intraoperative complications, and postoperative ICU stay than those without preoperative IABP use [30]. On this basis, we recognize that preoperative IABP implantation partly reflects a patient’s worse functional status and resistance to surgical and clinical support measures. However, there is study on report that preoperative IABP use reduces postoperative mortality in high-risk populations of patients undergoing CABG surgery [33], which in part underscoring the clinical benefit of preoperative IABP implantation. Our study found no significant difference in in-hospital mortality between patients in the Pro-LOIT and Nor-LOIT group, which may be explained by the lack of population stratification based on the risk.

Diabetes mellitus (DM) is a systemic disorder of glucose metabolism, characterized by insulin resistance, hyperglycemia, and hyperinsulinism, along with dyslipidemia. DM has been regarded as a risk factor for a variety of cardiovascular and cerebrovascular diseases, such as coronary heart disease, myocardial infarction, heart failure, ischemic and hemorrhagic cerebral infarction [34, 35]. Previous studies have investigated the associated risk factors of IABP insertion during CABG surgery and found that DM is an associated factor for intraoperative IABP implantation [36, 37]. Clinical studies have found that ACS patients with DM have higher risks of heart failure and short- and long-term mortality than ACS patients without DM [38]. We identified a promoting effect of diabetes on prolonged IABP implantation, which may be attributed to global cardiac function and the patient’s own resistance to disease. Additionally, despite the logistic regression model not considering blood glucose levels as a risk factor, patients in the Pro-LOIT group had higher fasting blood glucose levels than those in the Nor-LOIT group, consistent with the finding that diabetes was more common in the Pro-LOIT group. However, it should be noted that fasting plasma glucose levels were within the normal range in both groups, which may have been due to the use of glucose-lowering medications or insulin.

Created machine learning models in this study overcome complex relationship between various variables and display good performance in predicting the risk factors associated with prolonged LOIT. Importantly, the logistic regression model exhibited most excellent predictive ability. Furthermore, our findings showed that LOS, length of ICU, and hospitalization costs were significantly higher in the Pro-LOIT group than in the Nor-LOIT group. To extent degree, this suggests that early intervention based on machine learning model-identified risk factors for prolonged LOIT may help to improve these clinical outcomes in patients. However, it must be mentioned that more samples and data are required for supporting whether this clinical application is effective. Likewise, we ought to consider the existed limitations in this study. Firstly, the nature of our study was retrospective, which may have biased the results to some extent. Secondly, there is a lack of the external validation, which might affect the generalizability of our findings and models. Therefore, in future, the external data need to be used. Thirdly, it is unclear whether the constructed risk prediction model can be translated into actual clinical benefits for patients, so prospective, multicenter studies are needed to evaluate.

### Conclusion

Created machine learning models in this study were used for personalized prediction of prolonged LOIT in patients with CABG. Our results have revealed that the logistic regression model exhibits a good predictive performance and identifies the risk factors associated with prolonged LOIT. This may contribute to improving perioperative.

### Electronic supplementary material

Below is the link to the electronic supplementary material.

Supplementary Material 1

## Data Availability

The data supporting the findings of this study are available from the corresponding author upon reasonable request.

## Abbreviations

ALB: Albumin
ACS: Acute coronary syndrome
AMI: Acute myocardial infarction
AUC: Area under the receiver operating characteristic curve
BNP: B-type natriuretic peptide
BUN: Blood urea nitrogen
CABG: Coronary artery bypass grafting
CNB: Complement Naive Bayes
CK-MB: Creatine kinase isoenzyme
cTnT: Cardiac troponin T
CRRT: Continuous renal replacement therapy
GNB: Gaussian Naive Bayes
HDL-C: High-density lipoprotein cholesterol
HR: Heart rate
IABP: Intra-aortic balloon pump
ICU: Intensive care units
KNN: K-nearest neighbors
LDL-C: Low-density lipoprotein cholesterol
LOS: Length of hospital stay
LVEF: Left ventricular ejection fraction
LVFS: Left ventricular fraction shortening
LVDs: Left ventricular end-systolic diameter
LVDd: Left ventricular end-diastolic diameter
MLP: Multi-layer perceptron neural network
NYHA: New York Heart Association
SVM: Support vector machine
Scr: Serum creatinine
SHAP: Shapley Additive exPlanations
TG: Triglyceride
TC: Total cholesterol

## References

[1] Jeger RV, Radovanovic D (2008). Ten-year trends in the incidence and treatment of cardiogenic shock. Ann Intern Med.

[2] Thiele H, Akin I (2018). One-year outcomes after PCI strategies in cardiogenic shock. N Engl J Med.

[3] Thiele H, Akin I (2017). PCI strategies in patients with Acute myocardial infarction and cardiogenic shock. N Engl J Med.

[4] Thiele H, de Waha-Thiele S, Freund A, Zeymer U, Desch S, Fitzgerald S (2021). Management of cardiogenic shock. EuroIntervention: J EuroPCR Collab Working Group Interventional Cardiol Eur Soc Cardiol.

[5] Morici N, Marini C (2022). Intra-aortic balloon pump for Acute-on-chronic heart failure complicated by cardiogenic shock. J Card Fail.

[6] Thiele H, Zeymer U (2013). Intra-aortic balloon counterpulsation in acute myocardial infarction complicated by cardiogenic shock (IABP-SHOCK II): final 12 month results of a randomised, open-label trial. Lancet (London England).

[7] Thiele H, Zeymer U (2012). Intraaortic balloon support for myocardial infarction with cardiogenic shock. N Engl J Med.

[8] Ferguson JJ 3rd, Cohen M (2001). The current practice of intra-aortic balloon counterpulsation: results from the Benchmark Registry. J Am Coll Cardiol.

[9] Cohen M, Urban P (2003). Intra-aortic balloon counterpulsation in US and non-US centres: results of the Benchmark Registry. Eur Heart J.

[10] Parissis H, Soo A, Al-Alao B (2011). Intra aortic balloon pump: literature review of risk factors related to complications of the intraaortic balloon pump. J Cardiothorac Surg.

[11] Siriwardena M, Pilbrow A, Frampton C, MacDonald SM, Wilkins GT, Richards AM (2015). Complications of intra-aortic balloon pump use: does the final position of the IABP tip matter?. Anaesth Intensive Care.

[12] Boudoulas KD, Bowen T, Pederzolli A, Pfahl K, Pompili VJ, Mazzaferri EL (2014). Jr. Duration of intra-aortic balloon pump use and related complications. Acute Card Care.

[13] Fuernau G (2020). Impact of timing of intraaortic balloon counterpulsation on mortality in cardiogenic shock - a subanalysis of the IABP-SHOCK II trial. Eur Heart J Acute Cardiovasc care.

[14] Zhou M, Yu K (2017). Analysis on application timing of IABP in emergency PCI treatment of patients with combined acute myocardial infarction and cardiac shock. Eur Rev Med Pharmacol Sci.

[15] Cofre-Martel S, Lopez Droguett E, Modarres M. Big Machinery Data Preprocessing Methodology for Data-Driven Models in Prognostics and Health Management. Sensors (Basel, Switzerland). 2021; 21. 10.3390/s21206841 PMC8537368 34696058

[16] Erickson BJ (2021). Basic Artificial Intelligence techniques: Machine Learning and Deep Learning. Radiol Clin North Am.

[17] Yoon HK, Yang HL, Jung CW, Lee HC (2022). Artificial intelligence in perioperative medicine: a narrative review. Korean J Anesthesiol.

[18] Tseng PY, Chen YT (2020). Prediction of the development of acute kidney injury following cardiac surgery by machine learning. Crit Care.

[19] Park J, Bonde P (2022). Machine learning in cardiac surgery: Predicting Mortality and Readmission. ASAIO J (American Soc Artif Intern Organs: 1992).

[20] Mahajan A, Esper S (2023). Development and validation of a machine learning model to identify patients before surgery at high risk for postoperative adverse events. JAMA Netw Open.

[21] Li Q, Lv H (2024). Development and validation of a machine learning prediction model for perioperative red blood cell transfusions in cardiac surgery. Int J Med Informatics.

[22] Saxby DJ, Killen BA (2020). Machine learning methods to support personalized neuromusculoskeletal modelling. Biomech Model Mechanobiol.

[23] Graeßner M, Jungwirth B (2023). Enabling personalized perioperative risk prediction by using a machine-learning model based on preoperative data. Sci Rep.

[24] Jentzer JC, van Diepen S, Henry TD, Baran DA, Barsness GW, Holmes DR (2022). Influence of intra-aortic balloon pump on mortality as a function of cardiogenic shock severity. Catheterization Cardiovasc Interventions: Official J Soc Cardiac Angiography Interventions.

[25] Kusumoto G, Shigematsu K, Iwashita K, Tominaga K, Totoki T, Yamaura K (2017). Association between Preoperative Cardiac Left Ventricular Dysfunction and Perioperative Intraaortic Balloon pump in patients undergoing off-pump coronary artery bypass surgery. Heart Surg Forum.

[26] Barbagallo M, Casati A (2006). Early increases in cardiac troponin levels after major vascular surgery is associated with an increased frequency of delayed cardiac complications. J Clin Anesth.

[27] Nagele P, Brown F (2013). High-sensitivity cardiac troponin T in prediction and diagnosis of myocardial infarction and long-term mortality after noncardiac surgery. Am Heart J.

[28] Gualandro DM, Puelacher C (2018). Comparison of high-sensitivity cardiac troponin I and T for the prediction of cardiac complications after non-cardiac surgery. Am Heart J.

[29] Mokhtar AT, Begum J, Buth KJ, Legare JF (2017). Cardiac troponin T is an important predictor of mortality after cardiac surgery. J Crit Care.

[30] Deppe AC, Weber C (2017). Preoperative intra-aortic balloon pump use in high-risk patients prior to coronary artery bypass graft surgery decreases the risk for morbidity and mortality-A meta-analysis of 9,212 patients. J Card Surg.

[31] Kucuker A, Cetin L (2014). Single-centre experience with perioperative use of intraaortic balloon pump in cardiac surgery. Heart Lung Circ.

[32] Mannacio V, Di Tommaso L, De Amicis V, Stassano P, Musumeci F, Vosa C (2012). Preoperative intraaortic balloon pump for off-pump coronary arterial revascularization. Ann Thorac Surg.

[33] Escutia-Cuevas HH, Suárez-Cuenca JA (2020). Preoperative use of intra-aortic balloon pump support reduced 30-Day mortality in a Population with LVEF > 35% and High Surgical risk after coronary artery bypass graft surgery. Cardiology.

[34] Dal Canto E, Ceriello A (2019). Diabetes as a cardiovascular risk factor: an overview of global trends of macro and micro vascular complications. Eur J Prev Cardiol.

[35] Haratz S, Tanne D (2011). Diabetes, hyperglycemia and the management of cerebrovascular disease. Curr Opin Neurol.

[36] Ahmad I, Islam MU, Rehman MU, Khan B (2021). Frequency of intra-aortic balloon pump insertion and associated factors in coronary artery bypass grafting in a tertiary care hospital. Pakistan J Med Sci.

[37] Iyengar A, Kwon OJ (2018). Predictors of cardiogenic shock in cardiac surgery patients receiving intra-aortic balloon pumps. Surgery.

[38] Cavallari I, Cannon CP (2018). Metabolic syndrome and the risk of adverse cardiovascular events after an acute coronary syndrome. Eur J Prev Cardiol.

