# Supplementary figures and images for: Machine learning-based model development for predicting risk factors of prolonged intra-aortic balloon pump therapy in patients with coronary artery bypass grafting
Source: J Cardiothorac Surg. 2024 Jun 26;19:383. doi: 10.1186/s13019-024-02830-8 (PMC11201335; doi:10.1186/s13019-024-02830-8)

**Graphical abstract:**


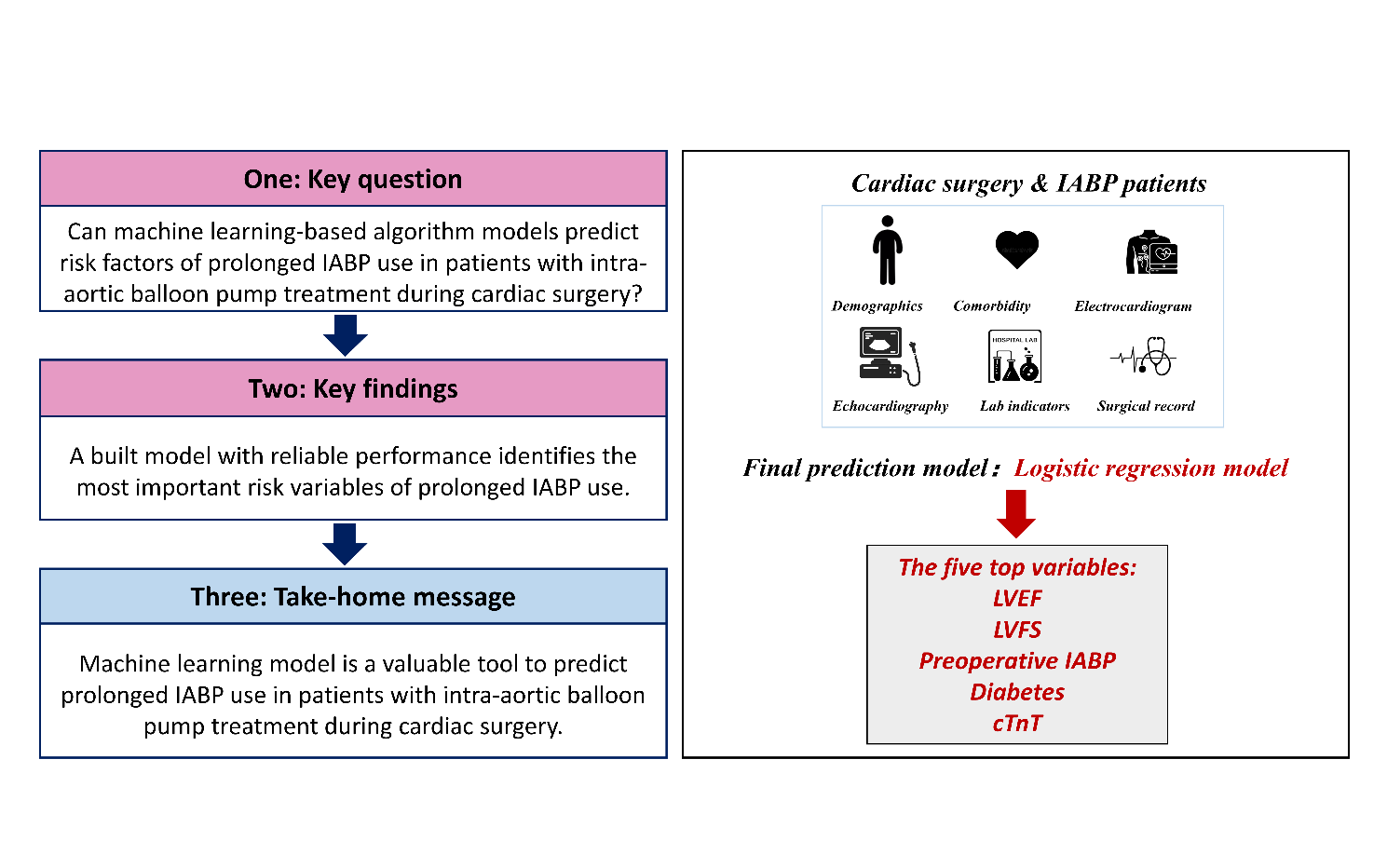

Supplement: Supplementary file 1 — Supplementary Material 1 [file 13019_2024_2830_MOESM1_ESM.docx]
